# Hydrogen Sulfide and Persulfides Oxidation by Biologically Relevant Oxidizing Species

**DOI:** 10.3390/antiox8020048

**Published:** 2019-02-22

**Authors:** Dayana Benchoam, Ernesto Cuevasanta, Matías N. Möller, Beatriz Alvarez

**Affiliations:** 1 Laboratorio de Enzimología, Instituto de Química Biológica, Facultad de Ciencias, Universidad de la República, Montevideo 11400, Uruguay; dbenchoam@fcien.edu.uy (D.B.); ecuevasanta@fcien.edu.uy (E.C.); 2 Center for Free Radical and Biomedical Research, Universidad de la República, Montevideo 11800, Uruguay; mmoller@fcien.edu.uy; 3 Unidad de Bioquímica Analítica, Centro de Investigaciones Nucleares, Facultad de Ciencias, Universidad de la República, Montevideo 11400, Uruguay; 4 Laboratorio de Fisicoquímica Biológica, Instituto de Química Biológica, Facultad de Ciencias, Universidad de la República, Montevideo 11400, Uruguay

**Keywords:** hydrogen sulfide, persulfide, hydropersulfide, reactive oxygen species, sulfiyl radical

## Abstract

Hydrogen sulfide (H_2_S/HS^–^) can be formed in mammalian tissues and exert physiological effects. It can react with metal centers and oxidized thiol products such as disulfides (RSSR) and sulfenic acids (RSOH). Reactions with oxidized thiol products form persulfides (RSSH/RSS^–^). Persulfides have been proposed to transduce the signaling effects of H_2_S through the modification of critical cysteines. They are more nucleophilic and acidic than thiols and, contrary to thiols, also possess electrophilic character. In this review, we summarize the biochemistry of hydrogen sulfide and persulfides, focusing on redox aspects. We describe biologically relevant one- and two-electron oxidants and their reactions with H_2_S and persulfides, as well as the fates of the oxidation products. The biological implications are discussed.

## 1. Hydrogen Sulfide: An Ancient Metabolite with Novel Regulatory Roles

Hydrogen sulfide (H_2_S) is the simplest molecule containing reduced sulfur among the wide diversity of sulfur compounds. It was first synthesized as a gas, and its composition was identified in the eighteenth century [1]. It is naturally occurring in geothermal sources [2] and has been related to the emergence and evolution of organisms in Earth [3]. It is a crucial molecule in the metabolism of several organisms, acting both as an electron donor in energetic pathways (anoxygenic photosynthesis and oxidative phosphorylation in lithotrophs [4]) and as a sulfur donor in synthetic pathways (cysteine and methionine synthesis, among others [5]). Furthermore, sulfur- and sulfate-reducing organisms are able to produce H_2_S in assimilative or dissimilative processes [6].

H_2_S was identified as a toxic compound for humans centuries ago. High levels of H_2_S can induce inhibition of cellular respiration [7]. Exposure of mammals to H_2_S from sewage, industrial sources and domestic accidents have produced numerous cases of loss of consciousness and death [8]. However, some animals are able to tolerate elevated levels of H_2_S and have efficient systems for its detoxification [9]. Indeed, in the human intestine, mitochondria are able to deal with the H_2_S produced by the enteric flora [10], which diffuses easily through membranes [11,12,13] and represents a hazard for neighboring tissues. The enterocytes are able to fuel the electronic transport chain by oxidizing H_2_S to sulfate (SO_4_^2–^) and thiosulfate (S_2_O_3_^2–^) [14]. The discovery of endogenous biogenesis of H_2_S in mammals [15,16] and the evidence of modulatory effects when administered at low concentrations led to the hypothesis of a regulatory role [17,18,19]. Numerous physiological responses to H_2_S have been reported since, including interactions with several cation channels in the nervous system, the attenuation of myocardial ischemia reperfusion injury, promotion of angiogenesis, and the induction of pro-/anti-inflammatory responses [20]. Several reports assigned an antioxidant role for H_2_S, which could be related to cytoprotective effects [21,22,23,24,25,26]; nevertheless, consideration of the kinetics of the reactions suggests that the protective role is not due to direct reactions of H_2_S with oxidants, as will be described below [27]. 

## 2. Persulfides as Potential Transducers of H_2_S Signaling 

The observed physiopathological effects focused the research in the field on the identification of cellular targets and on the underlying signaling mechanisms in order to develop plausible routes for pharmacological intervention. H_2_S shows nucleophilicity and is analogous to thiols, albeit weaker [27,28]. Among some possible targets in cells, H_2_S can react with metallic centers (iron, copper, zinc, molybdenum) producing coordination complexes and, in some circumstances, donating electrons. H_2_S is also able to react with oxidized forms of protein thiols such as disulfides (RSSR) or sulfenic acids (RSOH), thus leading to the formation of persulfides (RSSH/RSS^–^) [28,29]. 

The persulfides, which are also intermediates in the synthesis of particular sulfur biomolecules [30,31], are nowadays being considered candidate species for the transduction of the signals triggered by H_2_S. Not only are these species suggested to be more nucleophilic and acidic than the original thiols, but they also show an electrophilic character that is lacking in the original thiols [32]. In addition, enzymatic pathways for the H_2_S–independent formation of persulfides have been reported [33,34,35,36,37,38,39,40,41,42]. The generation of persulfides on critical protein cysteines constitutes a posttranslational modification which could affect relevant activities in cells [43,44] and thus allow for signal transduction and the modulation of processes. Furthermore, persulfides are able to react with oxidants. Direct and indirect protective effects of persulfides have been hypothesized and possible antioxidant roles are being surveyed [45,46,47,48].

The biochemistry of H_2_S and persulfides is now under intense scrutiny. In this work we describe the characteristics of these species from a redox biochemistry perspective, focusing on the reactions of H_2_S and persulfides with reactive species of biological interest. 

## 3. Chemistry and Biology of H_2_S

H_2_S is recognized by its odor of rotten eggs. Humans can detect as low as 0.0005–0.3 ppm, whereas concentrations higher than 10 ppm start to cause discomfort and higher than 500 ppm cause rapid loss of consciousness and death [49].

The recommended names by IUPAC are dihydrogen sulfide and sulfane for H_2_S, and sulfanide or hydrogen(sulfide)(1–) for HS^–^ [20]. In this review, “H_2_S” will be used for the gas and for the mixture of H_2_S and HS^–^ in aqueous solution. 

H_2_S may be considered at first sight to be similar to water, but there are several important differences that lead to dissimilar physical and chemical properties. Sulfur is larger than oxygen (van der Waals radii of 1.80 and 1.42 Å, respectively) [50], has a lower electronegativity (2.58 and 3.44 in the Pauling scale, respectively), and is more polarizable [51]. Hence, the dipole moment of H_2_S is lower than that of water, and hydrogen bonds in H_2_S are not formed at room temperature [51]; this explains why H_2_S is a gas at ambient temperature and pressure. H_2_S can dissolve in water with a relatively high solubility (101.3 mM/atm at 25 °C) [52], and the solvation is dominated by dispersive forces with no hydrogen bonds with water [51]. The slightly hydrophobic character of H_2_S is further supported by its twice higher solubility in organic solvents such as octanol and hexane than in water [12]. 

H_2_S is a weak diprotic acid with p*K*_a1_ = 6.98 at 25 °C and 6.76 at 37 °C (Equation 1) [53]. The second p*K*_a2_ has been more difficult to determine exactly but lies between 17 and 19 (Equation 2) in aqueous solution at 25 °C [20,53]. Therefore, S^2–^ concentrations will be very low in biological settings and will not contribute significantly to H_2_S reactivity. At pH 7.4 and 25 °C, total sulfide exists 28% as H_2_S, 72% as HS^–^, and as insignificant amounts of S^2–^.

H_2_S ⇌ HS^−^ + H^+^ (1)

HS^−^ ⇌ S^2−^ + H^+^ (2)

The hydrophobic character of H_2_S results in a higher solubility in membrane lipids than in water that results in a rapid diffusion through cell membranes without assistance by protein channels [11,12,13]. Permeability coefficients are estimated to be larger than 0.5 cm/s and probably larger than 10 cm/s [11,12,13], which indicates that membranes could slow down H_2_S diffusion 10–100 times compared to an equally thick layer of water. This is a low barrier that does not impede free diffusion across cellular membranes and only becomes significant when considering diffusion across a large number of membranes such as in a tissue [12].

The oxidation state of sulfur in H_2_S and in HS^–^ is –2. Thus, H_2_S can only be oxidized; it cannot act as an oxidant. Therefore, the statements in the literature regarding H_2_S oxidation of protein cysteines to persulfides are not correct; either the thiol or H_2_S need to have undergone previous oxidation for cysteine persulfide formation to occur. Oxidation of H_2_S can lead to various products in which the sulfur can have oxidation numbers up to +6. The oxidation products include sulfate (SO_4_^2^^−^), sulfite (SO_3_^2^^−^), thiosulfate (S_2_O_3_^2^^−^), persulfides (RSS^−^), organic (RSS_n_SR) and inorganic (HSS_n_S^−^) polysulfides, and elemental sulfur (S_n_). 

### 3.1. Biological Sources of H_2_S

Mammals obtain reduced sulfur from cysteine and methionine in the diet. The latter is an essential amino acid, but cysteine can be synthesized from methionine through the transsulfuration pathway. Two proteins involved in this metabolic pathway, cystathionine β-synthase (CBS) and cystathionine γ-lyase (also called γ-cystathionase, CSE), can produce H_2_S. Another enzyme from sulfur metabolism, 3-mercaptopyruvate sulfurtransferase (MST), also produces H_2_S [20,32]. 

The canonical reaction of CBS is the β-replacement of L-serine by L-homocysteine to generate L-cystathionine and H_2_O, but CBS also catalyzes other reactions using alternative substrates such as cysteine instead of serine that leads to cystathionine and H_2_S [54]. Mutations in this protein are the most common cause of hereditary homocystinuria [55]. CSE catalyzes the γ-elimination of cystathionine to give cysteine, α-ketobutyrate, and ammonia, but, like CBS, can also catalyze other reactions that lead to formation of H_2_S, such as the β-elimination of cysteine that is estimated to be the major reaction involved in H_2_S synthesis by CSE [54]. MST catalyzes the transfer of the sulfur atom from 3-mercaptopyruvate to a nucleophilic acceptor such as thioredoxin or glutathione (GSH), which subsequently releases H_2_S [56]. 

There are some differences in tissue distribution and subcellular localization. CSE is considered a cytosolic protein that predominates in cardiovascular tissue and liver; CBS is also cytosolic and is considered an important source of H_2_S in the nervous system; MST has been found both in the cytosol and the mitochondria and is the other important source of H_2_S in the nervous system [20]. 

### 3.2. Biological Sinks of H_2_S

There are several mechanisms that decompose H_2_S. The main pathway for H_2_S detoxification is located in the mitochondria and involves oxidation of sulfide to sulfate and thiosulfate [20]. The enzyme sulfide quinone oxidoreductase (SQR) oxidizes H_2_S and reduces coenzyme Q. An intermediate SQR-persulfide is formed and transferred either to glutathione to make glutathione persulfide (GSSH) or to sulfite to form thiosulfate. GSSH is subsequently metabolized by persulfide dioxygenase (PDO, also called ethylmalonic encephalopathy protein 1, ETHE1) to glutathione and sulfite (SO_3_^2–^), and the latter is oxidized to sulfate (SO_4_^2–^) by sulfite oxidase. Rhodanese could use GSSH and sulfite to make thiosulfate. Alternatively, thiosulfate could be utilized by rhodanese to produce GSSH [57,58,59].

H_2_S also reacts with ferric hemoglobin and myoglobin (methemoglobin and metmyoglobin, respectively) through a complex mechanism that results in the formation of thiosulfate and iron-bound polysulfides [60,61]. Though the proportion of methemoglobin is usually below 1% [62], the concentration of total hemoglobin subunits in red blood cells is 20 mM, and thus up to 200 µM methemoglobin may be available to react with H_2_S. Besides, several medications and diseases are known to increase methemoglobin [62], so this may be an important sink for H_2_S in circulation. Furthermore, in the presence of oxygen or hydrogen peroxide, H_2_S can modify hemes covalently in these globins and some other hemeproteins, thus forming green sulfheme in which the sulfur is covalently bound to one pyrrole ring. These processes will be further analyzed in Section 4.2.6. 

### 3.3. Physiology and Pharmacology of H_2_S

Although early studies indicated that physiological concentrations of H_2_S in different tissues were in the 30–100 µM range, improved detection methods suggest that actual concentrations are in the 4–55 nM range for most tissues [63,64,65,66].

Externally added H_2_S at higher concentrations has been observed to have different effects. In the cardiovascular system, H_2_S has multiple nitric oxide-dependent and independent effects that include the relaxation of smooth muscle cells, regulation of blood pressure, promotion of angiogenesis, and attenuation of myocardial ischemia reperfusion injury [19,67,68,69,70,71,72]. In the nervous system, H_2_S is considered to be a neuromodulator. For example, it affects hippocampal long-term potentiation [18]. It has also been shown to inhibit HOCl- and peroxynitrite-mediated toxicity in neuroblastoma cells [21,23]. Moreover, H_2_S protects neurons from oxidative stress by increasing γ-glutamylcysteine synthetase activity and cystine transport, which induces the production of reduced glutathione [22]. In the respiratory system, H_2_S has potential therapeutic uses against fibrosis and chronic obstructive pulmonary disease [73,74]. In the liver, a hepatoprotective effect of H_2_S has been observed against ethanol-induced liver injury in mice [75]. 

H_2_S has shown both pro- and anti-inflammatory effects. In sepsis and acute pancreatitis, a pro-inflammatory effect of H_2_S has been observed [76,77,78], whereas an anti-inflammatory effect has been reported for intestinal ischemic damage and ethanol-induced gastritis [79,80,81]. 

The antioxidant effects cannot be attributed to direct reactions with oxidants since, from a kinetic perspective, H_2_S may not be able to trap oxidative species, as rationalized below. The indirect mechanisms underlying the observed antioxidant effects may be various. For example, H_2_S was proposed to inhibit oxidative stress through the persulfidation of Keap1, which activates nuclear factor (erythroid-derived 2)-like 2 (Nrf2) signaling and promotes its translocation to the nucleus and the expression of antioxidant pathways [75,82,83,84,85].

## 4. Oxidizing Species in Biology and Their Reactions with H_2_S

The formation of partial oxygen reduction products can occur in biological systems. These free radical and non-free radical species are superoxide radical (O_2_•^–^), hydrogen peroxide (H_2_O_2_), and hydroxyl radical (HO•). Together with secondary species such as peroxynitrite (ONOO^–^), hypochlorous acid (HOCl), or organic peroxyl radicals (ROO•), they are in general called reactive oxygen species (ROS); however, this term should be used with caution because it gives the erroneous idea that there is only one ill-defined species that oxidizes all biomolecules. In contrast, the reactivity of the oxidants varies, and their targets in biological settings are determined by kinetic aspects of rate constants multiplied by the concentration of the potential target as well as compartmentalization and membrane permeability aspects. The different species can be divided into two-electron or one-electron oxidants. The latter are often free radicals that start oxygen-dependent chain reactions. 

One important site of reactive species formation is the mitochondrial electron transport chain. They can also be formed by ionizing and UV radiation, through the redox cycling of certain xenobiotics, and enzymatically through the action of NADPH oxidases, xanthine oxidase, hemeperoxidases, and cytochromes P450. Nitric oxide synthase generates nitric oxide, which, by itself, is not highly oxidizing, but it can react with superoxide radical generating peroxynitrite and secondary oxidants derived from it. 

The high reactivity of some of these species with biomolecules can lead to cell damage; this aspect is exploited by immune cells in their warfare against microorganisms. Oxidant action can be counteracted, in some cases, by antioxidants; for example, in the free radical process of lipid peroxidation, peroxyl radical intermediates can be scavenged by the antioxidant vitamins E and C. In addition to their cell damaging action, oxidants can have signaling roles, as becomes clear from the evidence for the actions of hydrogen peroxide as a second messenger [86]. Thus, the classical concept of oxidative stress as a disbalance between oxidant formation and antioxidant action has been extended to include the concept of a disbalance in regulatory pathways. 

**Scheme 1:**
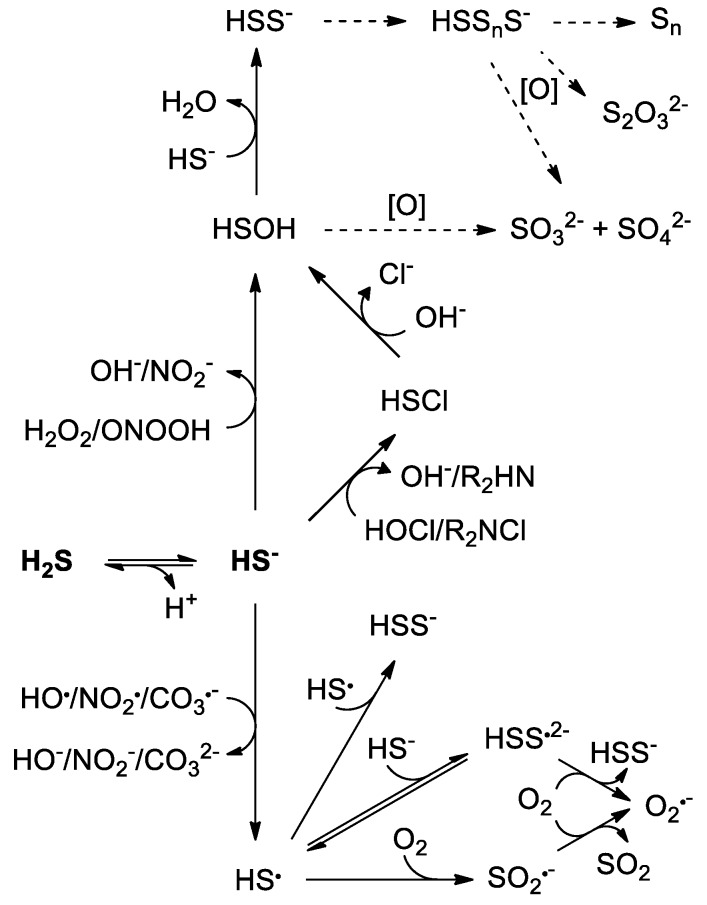
Hydrogen sulfide oxidation by reactive species. The dashed lines represent processes that are accomplished in more than one step. Protons are sometimes omitted for simplicity.

In the following paragraphs, we briefly describe the oxidants potentially involved in the two- and one-electron oxidation of H_2_S, we provide the rate constants of the reactions, and we discuss the fate of some of the products formed. The reactions are summarized in Scheme 1.

### 4.1. Two-Electron Oxidation

H_2_S is a strong reductant according to the two-electron reduction potential *E°’*(HSS^–^, H^+^/2HS^–^) = –0.23 V [87,88]. The disulfide HSSH/HSS^–^ is not formed directly but in at least two steps. Rather, sulfenic acid (HSOH) is formed, an unstable intermediate that reacts fast with a second H_2_S to form HSSH/HSS^–^. The latter is also unstable and can disproportionate yielding polysulfides of different chain lengths, elemental sulfur and H_2_S. Polysulfides can also react with reductants or be oxidized to oxyacids. Several two-electron oxidants can react with H_2_S.

#### 4.1.1. Hydrogen Peroxide

This two-electron oxidant (*E°’*(H_2_O_2_, 2H^+^/2H_2_O) = +1.35 V) [89] is formed mainly by the dismutation of superoxide radical, which can be spontaneous or catalyzed by superoxide dismutases. It can also be formed directly by some oxidases. Though it is a strong oxidant, hydrogen peroxide reacts relatively slowly with most biomolecules, with the exception of peroxidases containing selenocysteine, cysteine or heme. Accordingly, the reaction between H_2_S and hydrogen peroxide has a rate constant of 0.73 M^–1^ s^–1^ at pH 7.4 and 37 °C [27]. The primary product is HSOH. The final products depend on the initial ratios of hydrogen peroxide to H_2_S and consist mainly of polysulfides, elemental sulfur and, in the presence of excess oxidant, sulfate. Curiously, the system can behave like a chemical oscillator [90,91].

#### 4.1.2. Hypochlorous Acid and Chloramines 

Hypochlorous acid (HOCl) is formed from the oxidation of chloride ions by the neutrophil enzyme myeloperoxidase. HOCl is a highly oxidizing species (*E°’*(HClO, H^+^/Cl^–^, H_2_O) = +1.28 V) [92] and reacts fast with HS^–^ with a second order rate constant of 0.8–20 × 10^8^ M^–1^ s^–1^ at pH 7.4 [27,93]. Hypochlorous acid usually reacts by transferring a Cl^+^ species to a nucleophile. Thus, the reaction with HS^–^ is likely to proceed through the formation of HSCl, which quickly hydrolyzes to HSOH. The hypochlorite ion is in equilibrium with hypochlorous acid (p*K*_a_ 7.47). Hypochlorite can also react with HS^–^, albeit several orders of magnitude more slowly [93]. At an excess of H_2_S with respect to hypochlorous acid, the main product formed is elemental sulfur, with the intermediacy of polysulfides [93].

The reaction of hypochlorous acid with amines leads to chloramines (RHNCl and R_2_NCl). These are less reactive and more selective oxidants than hypochlorous acid. Taurine chloramine reacts with H_2_S with a rate constant of 3 × 10^2^ M^–1^ s^–1^ at pH 7.4 and 37 °C [27].

#### 4.1.3. Peroxynitrite

This anion (ONOO^–^) and its conjugate acid, peroxynitrous acid (ONOOH, p*K*_a_ 6.8), are formed from the reaction of superoxide radical with nitric oxide, which occurs at nearly diffusion controlled rates. Peroxynitrous acid is unstable and can decay in buffer to nitric acid, hydroxyl radical, and nitrogen dioxide. *In vivo*, peroxynitrite can either react directly with targets such as thiols or metal centers, or it can react with carbon dioxide, leading to the formation of the secondary radicals, nitrogen dioxide and carbonate radical [94]. Peroxynitrous acid reacts directly with HS^–^ with a second order rate constant of 6.7 × 10^3^ M^–1^ s^–1^ (pH 7.4, 37 °C) [95] and, after a short lag, yellow products are formed [96]. According to computational simulations, the process starts with the nucleophilic substitution of HS^–^ on ONOOH to give HSOH and NO_2_^–^ as initial products. In the presence of excess H_2_S, HSOH reacts with a second HS^–^ to yield HSS^–^/HSSH. It was proposed that the yellow products originate from the secondary reaction of HSS^–^/HSSH with peroxynitrite, and that at least one of the yellow products is HSNO_2_ or its isomer HSONO [95]. 

In addition to the direct reaction of peroxynitrous acid with HS^–^, the free radicals derived from peroxyinitrite decay can also react with H_2_S and trigger oxygen dependent chain reactions that can be evidenced, for example, by the consumption of oxygen [27].

### 4.2. One-Electron Oxidation

The one-electron reduction potential of H_2_S is (*E°’*(HS•, H^+^/H_2_S) = +0.91–0.94 V) [87,97]. This value means that, in principle, only relatively strong one-electron oxidants such as hydroxyl radical (HO^•^), carbonate radical (CO_3_•^–^), nitrogen dioxide (NO_2_•), and peroxidase oxoferryl compounds I and II can oxidize H_2_S to HS•/S•^–^. However, further reactions of HS•/S•^–^ can drive the oxidation processes, extending the list of possible initial oxidants to, for example, metal centers with lower one-electron reduction potentials than that of the HS•/H_2_S couple. 

The initial product of the one-electron oxidation of H_2_S is the sulfiyl radical (HS•/S•^–^). This is an oxidizing radical and can react with several electron or hydrogen atom donors (AH, Equation 3). In addition, it can react with a second HS•/S•^–^ radical and form HSSH/HSS^–^ (Equation 4). Alternatively, HS•/S•^–^ can react with HS^–^ to form HSS•^2–^, a reducing radical that can react with oxygen forming superoxide radical (Equations 5,6). HS•/S•^–^ can also react with oxygen and form SO_2_•^–^, which can again react with oxygen to form superoxide radical (Equations 7,8). The latter dismutates to hydrogen peroxide and oxygen, which helps to drive the process.

HS•/S•^−^ + AH → H_2_S/HS^−^ + A•
(3)HS•/S•^−^ + HS•/S•^−^ → HSSH/HSS^–^   *k* = 6.5 × 10^9^ M^–1^ s^–1^ [98](4)HS•/S•^−^ + HS^−^ ⇌ HSS•^2−^
*k*_f_ = 5.4 × 10^9^ M^–1^ s^–1^; *k*_r_ = 5.3 × 10^5^ s^–1^ [98]   (5)HSS•^2–^ + O_2_ → HSS^–^ + O_2_•^−^
*k* = 4 × 10^8^ M^–1^ s^–1^ [98](6)HS•/S•^−^ + O_2_ → SO_2_•^−^
*k* = 7.5 × 10^9^ M^–1^ s^–1^ [98](7)SO_2_•^−^ + O_2_ → SO_2_ + O_2_•^−^
*k* = 1 × 10^8^ M^–1^ s^–1^ [99](8)

In addition to these relatively well characterized reactions, several other reactions can occur, which lead to a wide variety of possible radical and non-radical products and unstable intermediates. It is clear that the one-electron oxidation of H_2_S can trigger oxygen-dependent free radical chain reactions that can lead to the amplification of initial oxidation events. Several one-electron oxidants can initiate these events. 

#### 4.2.1. Oxygen

The one-electron oxidation of H_2_S by oxygen (O_2_) is thermodynamically uphill, since the *E°’*(O_2_/O_2_•^–^) = −0.35 V (–0.18 V for a 1 M O_2_ standard state) [89]. It is also very slow because of the spin barrier imposed by the diradical character of O_2_. Nevertheless, H_2_S autoxidation occurs and is a major concern in practical work with H_2_S. The process is likely driven by the reactivity of the initial products, HS• and O_2_•^–^, as well as that of secondary intermediates. Autoxidation is very slow and dependent on pH. It is critically affected by the presence of trace metal ions that can act as catalysts. It is also mechanistically complex due to the involvement of radical and non-radical intermediates. The products formed are usually elemental sulfur (S_n_), sulfate (SO_4_^2–^), sulfite (SO_3_^2–^), and thiosulfate (S_2_O_3_^2–^) [100,101].

#### 4.2.2. Superoxide Radical

This species is the primary free radical formed from the partial reduction of oxygen. Superoxide radical (O_2_•^–^) can dismutate to oxygen and hydrogen peroxide, spontaneously or enzymatically. It can also react with nitric oxide yielding peroxynitrite. This radical can be both oxidizing and reducing, with *E°’*(O_2_•^–^, 2H^+^/H_2_O_2_) = +0.91 V and *E°’*(O_2_/O_2_•^–^) = –0.18 V (1 M O_2_ standard state) [89]. It can react with H_2_S with a rate constant of ~208 M^–1^ s^–1^ in dimethyl sulfoxide [102]. However, rather than being scavenged by H_2_S, superoxide radical can be formed in H_2_S oxidation processes due to free radical chain reactions, as described in Section 4.2. 

#### 4.2.3. Hydroxyl Radical

The highly oxidizing hydroxyl radical (HO•) is formed in biological systems mainly through the Fenton reaction, i.e., through the reduction of hydrogen peroxide to HO• and H_2_O by reduced metal ions (Fe^2+^ or Cu^+^). With a reduction potential of +2.31 V at pH 7 [89], hydroxyl radical is so oxidizing that it can react with most biomolecules, and the rate constants are close to the diffusion limit. It can react very fast with both H_2_S and HS^–^, according to Equations 9,10 [98,103].

HO• + H_2_S → H_2_O + HS•*k* = 1.1 × 10^10^ M^–1^ s^–1^ [103]  (9)HO• + HS^−^ → HSOH•^−^ → S•^−^/HS• + H_2_O/OH^−^  *k* = 5.4 × 10^9^ M^–1^ s^–1^ [103]  (10)

#### 4.2.4. Nitrogen Dioxide

This free radical (NO_2_•) can be formed from the autoxidation of nitric oxide, from the decay of peroxynitrite in the presence of carbon dioxide and from the oxidation of nitrite. It is an oxidizing species with *E°’*(NO_2_•/NO_2_^–^) = +1.04 V [88]. The reaction between H_2_S and nitrogen dioxide was studied by pulse radiolysis. The rate constant is 3 × 10^6^ M^–1^ s^–1^ at pH 6 (25 °C) and increases at more alkaline pH, suggesting that HS^–^ is the reacting species [27].

#### 4.2.5. Carbonate Radical

The formation of carbonate radical (CO_3_•^–^) depends mainly on the decay of peroxynitrite in the presence of carbon dioxide. It also depends on the oxidation of carbon dioxide/bicarbonate by the peroxidase activity of CuZn superoxide dismutase or by xanthine oxidase [94]. Carbonate radical is a strong one-electron oxidant (*E°’*(CO_3_•^–^, H^+^/HCO_3_^–^) = +1.77 V) [88]. It reacts with H_2_S with a rate constant of 2.0 × 10^8^ M^–1^ s^–1^ (pH 7, 20 °C) [97].

#### 4.2.6. Metal Centers

Ferric hemeproteins with an open sixth coordination position, such as methemoglobin, metmyoglobin, myeloperoxidase, and catalase, can oxidize H_2_S [60,61,104,105,106].

Depending on the protein, either protonated H_2_S or HS^–^ can bind to ferric heme [107]. The binding is stable enough in some hemeproteins to suggest a function in H_2_S storage and transport, as in hemoglobin I from *Lucina pectinata* [108]. In the case of methemoglobin and metmyoglobin, H_2_S binds quickly and reversibly to ferric heme. Deprotonation leads to a ferric-HS^–^ complex that can slowly evolve to ferrous-bound HS•. The latter species can react with a second H_2_S or with oxygen, which leads to the formation of polysulfides and thiosulfate as products [60,61,107,109,110].

Remarkably, the hexacoordinated protein ferric cytochrome *c* can also oxidize H_2_S. The process is mediated by reducing species derived from the HS•/S•^–^ radical [111].

The oxoferryl compounds I and II of myeloperoxidase are formed in the peroxidase cycle of the enzyme and have reduction potentials of +1.35 and +0.97 V [92]. They can react with H_2_S with rate constants of 1 × 10^6^ M^–1^ s^–1^ and 2.0 × 10^5^ M^–1^ s^–1^, respectively (pH 7.4 and 25 °C). Ferric myeloperoxidase can also oxidize H_2_S and is reduced to the ferrous state [105]. Under aerobic conditions, compound III (ferric-superoxide or ferrous-dioxygen complexes in resonance) is formed. This compound slowly evolves to the ferric enzyme, allowing the enzyme to turnover. Myeloperoxidase-catalyzed oxidation of H_2_S leads to the formation of polysulfides and protein persulfides [106].

In many cases, the interaction between a hemeprotein and H_2_S leads to the formation of sulfheme, where one of the pyrroles in the porphyrin ring is covalently modified with a sulfur atom. Such modification has been reported for some globins, lactoperoxidase, and catalase [112]. Many details need to be filled in regarding the mechanism of sulfheme formation. An adequately positioned histidine in the distal heme site has been proposed to be critical [112]. It has also been proposed that, in the case of myeloperoxidase, the presence of a sulfonium ion linkage between a methionine and the heme ring determines that, in contrast to lactoperoxidase, the sulfheme cannot be formed [106]. Computational simulations suggest that sulfheme formation occurs through a concerted interaction between the distal histidine, H_2_S, and a ferric-bound hydroperoxide to form oxoferryl compound II, H_2_O and HS•; the latter subsequently adds to a specific pyrrole site [113].

In addition to hemeproteins, CuZn and Mn superoxide dismutases are able to catalyze the oxidation of H_2_S by oxygen [114,115].

### 4.3. Other Oxidants

Along with the species described above, other oxidants of biological relevance are likely to react with H_2_S, by analogy to their reactions with thiols. Among these other oxidants, organic hydroperoxides, other hypohalous acids such as HOBr and HOSCN, and free radicals such as peroxyl and phenoxyl radicals are to be considered. 

### 4.4. Nitric Oxide

The crosstalk between nitric oxide and H_2_S is complex and can give rise to species that contain both S and N atoms. Among these, thionitrous acid (HSNO) and perthionitrite (S_2_NO^−^) have received particular attention [20,116,117,118,119,120,121]. 

### 4.5. Biological Implications of H_2_S Oxidation by Reactive Species

At the physiological pH of 7.4, the rate constants of the reactions of H_2_S with oxidants have similar values as those of typical biological thiols such as cysteine and glutathione [27]. The pH-independent rate constants corresponding to the HS^–^ species are lower than those of the thiolates species (RS^–^), probably due to the lack of the inductive effect of the methylene. However, the higher acidity of H_2_S with respect to thiols (p*K*_a_ of 6.98 for H_2_S compared to 8.29 for cysteine and 8.94 for glutathione [53,122]), determines a higher availability of the ionized species. The net effect is that the apparent rate constants at physiological pH are comparable. 

To analyze whether H_2_S can be a scavenger of oxidant species, the rates instead of the rate constants need to be taken into account, i.e., the products of rate constants times concentration. While the rate constants are similar to those of thiols, the concentration of H_2_S in tissues is considered to be submicromolar and several orders of magnitude lower than that of thiols, which is millimolar, so H_2_S cannot compete with thiols for oxidants. Similar considerations apply for the comparison between H_2_S and other oxidant scavengers such as ascorbic acid. Hence, H_2_S cannot be considered a typical antioxidant scavenger except under exceptional situations that lead to high concentrations such as exogenous administration together with enhanced oxidant production. Other mechanisms are probably underlying the observed antioxidant effects of H_2_S; for example, interference with signaling pathways. Furthermore, the one-electron oxidation of H_2_S can trigger free radical chain reactions, opening the possibility for pro-oxidant effects of H_2_S. 

## 5. Chemistry and Biology of Persulfides 

Persulfides (RSSH/RSS^–^) are sulfane sulfur compounds, meaning that they have a sulfur atom bound to two sulfurs or to a sulfur and an ionizable hydrogen [123]; the outer sulfur is the sulfane sulfur according to this criterion. RSSH can also be referred to with the terms hydropersulfide or hydrodisulfide. In this text, “persulfide” is used to designate mixtures of RSSH and RSS^−^. Their chemical properties differ from those of thiols; hydropersulfides are more acidic and can either act as nucleophiles when deprotonated (RSS^–^) or electrophiles when protonated (RSSH). They are formed in biological systems through H_2_S-dependent and independent pathways. It is worth noting that the reaction between H_2_S and thiols does not occur in the absence of an oxidant.

### 5.1. Reactivity of Persulfides

#### 5.1.1. Persulfide Acidity

Protonated persulfides ionize in water and form the corresponding anionic persulfides (Equation 11). Their acidity is predicted to be higher than that of the analogous thiols. Thus, at physiological pH, the anionic species would predominate. For instance, the p*K*_a_ of 2-[(3-aminopropyl)amino]ethane persulfide was estimated to be 6.2 ± 0.1, while the p*K*_a_ of the analogous thiol is 7.6 ± 0.1 [124].

RSSH ⇌ RSS^−^ + H^+^ (11)

#### 5.1.2. Persulfides as Nucleophiles

Anionic persulfides can act as nucleophiles (Equation 12). This is evidenced by their reactions with thiol alkylating agents such as monobromobimane and iodoacetamide, which yield disulfides. This is also evidenced by their reactions with disulfides such as 5,5-dithiobis(2-nitrobenzoic acid) and 4,4-dithiodipyridine to produce trisulfides [20,28,33,125,126,127,128,129]. Moreover, persulfides react with two- and one-electron oxidants as discussed in Section 6.

RSS^−^ + E^+^ → RSSE (12)

Persulfides have been proposed to have an increased nucleophilicity compared to analogous thiolates [126,130]. This can be explained by the alpha effect, namely, the increased reactivity of an atom due to the presence of unshared pairs of electrons in the adjacent atom [131,132]. This effect has been observed in the nucleophilicity of HOO^–^ compared to HO^–^ and NH_2_NH_2_ and NH_2_OH compared to NH_3_ [131]. The first determination of the alpha effect in persulfides was observed in human serum albumin, in which persulfide has an increased reactivity towards the disulfide 4-4’-dithiodipyridine compared to the thiolate derivative [28]. The alpha effect, together with the predominance of RSS^–^ versus RSSH at physiological pH due to the relatively high acidity, make persulfides better nucleophiles than thiols.

#### 5.1.3. Persulfides as Electrophiles

Protonated persulfides are weak electrophiles. They can react with nucleophiles to give thiols when the outer sulfur is attacked (Equation 13) or H_2_S when the inner sulfur is attacked (Equation 14). Whether the attack is on one sulfur or the other appears to be determined by steric hindrance [133,134] and by the acidity of the leaving group, with the better leaving group being the one with lower p*K*_a_. Hence, as low molecular weight (LMW) thiols have higher p*K*_a_s than H_2_S, i.e., the p*K*_a_ of cysteine is 8.29 [122] and the p*K*_a_ of H_2_S is 6.98 [53], the attack on the inner sulfur would predominate. In protein persulfides formed in acidic cysteines, the attack on the outer sulfur would predominate and restore the reduced protein.

RSSH + Nu^−^ → RS^−^ + NuSH (13)

RSSH + Nu^−^ → RSNu + HS^−^ (14)

The outer sulfur can react with cyanide, thiolates, sulfite [133,135], amines [136], hydroxide [137], and tertiary phosphines [138,139]. Persulfides and cyanide react to form the corresponding thiol and thiocyanate, which, in the presence of ferric ions, forms a colored complex that can be quantified spectrophotometrically. This is the basis of the method of cold cyanolysis used to quantify persulfides [123,140]. 

In the reaction of a persulfide with a thiol, the attack on the outer sulfur constitutes a transpersulfidation reaction. In addition to forming thiol, the sulfane sulfur is transferred and a new persulfide at the attacking thiol is formed (Equation 15). The reactions between persulfides and thiols are plausible to occur *in vivo*. Intermediate persulfides formed in the proteins MST, SQR, and rhodanese can be transferred to several acceptors including cyanide, sulfite, and thiols [34,57,58,141,142,143,144]. Cysteine desulfurases, such as those that participate in the synthesis of iron sulfur clusters, are another example of enzymes transferring the sulfane sulfur to a thiol in specific compounds [145]. The reduction of protein persulfides by thiols could constitute a protective mechanism to avoid irreversible cysteine modifications under oxidative stress [45,111,146]. A depersulfidase role for thioredoxin has been suggested. One of the proposed mechanisms involves the transfer of the sulfane sulfur from either LMW or protein persulfides to the nucleophilic cysteine of thioredoxin, which regenerates the original thiol and forms thioredoxin persulfide. Subsequently, H_2_S and oxidized thioredoxin are formed [29,147,148].

On the other hand, when the inner sulfur of a persulfide is attacked by a thiol, H_2_S is released and a mixed disulfide is formed (Equation 16) [125,139,149]. This mechanism has been suggested for the activation of Nrf2 by Keap1. Keap1 persulfide/Nrf2 was proposed to react with a protein thiolate to form a mixed disulfide and release H_2_S and Nrf2, inducing its activation to protect neuroblastoma cells from methylglyoxal-induced toxicity [150].

RSSH + R’S^−^ ⇌ RS^−^ + R’SSH (15)

RSSH + R’S^−^ ⇌ RSSR’ + HS^−^ (16)

#### 5.1.4. Disproportionation of Persulfides

Persulfides are highly unstable in aqueous solution, making their study difficult. This is related to their nucleophilic and electrophilic character that facilitate the reactions between them causing spontaneous decay through a disproportionation process. Depending on which sulfur is attacked, different products can be formed. The attack on the outer sulfur generates thiol and hydropolysulfides that evolve to elemental sulfur (Equations 17,18), while the attack on the inner sulfur yields polysulfides and H_2_S (Equation 19) [137]. The decay products are also likely to depend on steric effects and on the acidity of the leaving groups. For example, cysteine persulfide predominantly decays through the release of H_2_S instead of cysteine, which has a higher p*K*_a_ [35].

RSSH + RSS^−^ ⇌ RSSS^−^ + RSH (17)

RSSSH → RSH + S_n_ (18)

RSSH + RSS^−^ ⇌ RSSSR + HS^−^ (19)

### 5.2. Biological Sources of Persulfides

Several processes can give rise to persulfides in biological context; some are dependent on H_2_S and some are not. These processes are described below. In addition, it was recently proposed that cysteine persulfide can be generated from cysteine via cysteinyl-tRNA synthetase [41].

#### 5.2.1. Radical Processes

Sulfiyl (HS•) and thiyl radicals (RS•) could be formed by the reaction of strong one-electron oxidants and H_2_S or RSH, respectively. These radicals could rapidly react yielding a persulfide (Equation 20). Nevertheless, this reaction is not expected to be relevant *in vivo* due to the low concentration of radical species. A more likely radical pathway to form persulfides is the formation of the reducing radical RSSH•^–^ via the reaction of either HS• and RS^–^ or RS• and HS^–^ and the subsequent reaction with oxygen (Equations 21–23) [20,151].

HS• + RS• → RSSH(20)

HS• + RS^−^ → RSSH•^−^ (21)

RS• + HS^−^ → RSSH•^−^ (22)

RSSH•^−^ + O_2_ → RSSH + O_2_•^−^ (23)

#### 5.2.2. H_2_S and Oxidized Thiols

The reactions of H_2_S with oxidized thiols constitute biologically significant pathways for the formation of persulfides. H_2_S can react with disulfides releasing thiol (Equation 24) or with sulfenic acid eliminating water (Equation 25).

HS^−^ + RSSR’ ⇌ RSSH + R’S^−^ (24)

HS^−^ + RSOH → RSSH + OH^−^ (25)

Protein persulfides are formed endogenously through these pathways. For example, a persulfide intermediate of SQR is formed by the reaction of H_2_S with the active site disulfide of the enzyme. This reaction is ~10^6^ fold accelerated compared to the reaction with cystine [57,58,152]. In the endoplasmic reticulum, an environment with high concentration of disulfides, the reaction of H_2_S with disulfides could be more relevant than in compartments with scarce disulfides like the cytosol [151]. In cultured cells, an increase in persulfide concentration was observed after treatment with H_2_O_2_ that led to an increase in sulfenic acid and disulfide. However, this was not observed upon treatment with diamide that only increased disulfide concentration, which suggests that persulfide was formed by H_2_S and sulfenic acid [28,29]. The reaction of H_2_S with sulfenic acids could be a mechanism of protection against its overoxidation to sulfinic acid (RSO_2_H) and sulfonic acid (RSO_3_H), which are irreversible modifications. On the contrary, the oxidation of persulfidated residues leads to RSSO_2_H and RSSO_3_H species that can be reduced to thiols restoring the protein [20,29,45,46,153].

The reactions of H_2_S with disulfides and sulfenic acids are common strategies to prepare persulfides in the laboratory. Mixtures of LMW disulfides and H_2_S have been used as sources of persulfides [28,66,126,154,155]. However, the facts that reaction (24) is reversible and that the persulfides formed can react with disulfides to form trisulfides and thiols, need to be taken into account [128,129]. The kinetics of the reactions between H_2_S and LMW disulfides such as cystine, glutathione disulfide, homocystine, and 5,5’-dithiobis-(2-nitrobenzoic acid) to form LMW persulfides are slower than the reactions of thiols with disulfides [28]. The incubation of H_2_S and mixed disulfides of protein cysteines and acidic LMW thiols has been used to produce persulfides in papain [126], glutathione peroxidase Gpx3 [125], and albumin [28]. The reaction of protein sulfenic acid with H_2_S has also been used to prepare persulfides in albumin [28,151].

#### 5.2.3. Thiolates and Oxidized Sulfur Derivatives

Thiolates and oxidized sulfur derivatives of H_2_S react to form persulfides. As described previously, the attack of thiolates on the outer sulfur of persulfides produces a new persulfide at the attacking thiolate (Equation 15). For example, the persulfide intermediate formed in SQR has been proposed to react with different molecules. When it reacts with a thiol, a new persulfide is generated [42,58]. In addition, organic and inorganic polysulfides (RSS_n_SR, RSS_n_S^−^, HSS_n_S^−^) can be sources of persulfides. A few polysulfide-containing proteins have been detected, such as SOD1, IL-6, and IgG2 [156]. Recently, the reaction between dibenzyl trisulfide and tetrasulfide with cysteine and glutathione was shown to release H_2_S with a brief lag-time that was attributed to the formation of persulfides as intermediates [157]. Cysteine trisulfide treatment of cultured cells led to an increase in intracellular cysteine and glutathione persulfides [129]. 

#### 5.2.4. Elimination of Disulfide

The disulfide cystine in the presence of the pyridoxal phosphate-dependent enzymes, CBS and CSE, forms cysteine persulfide, ammonia, and pyruvate. In addition, CSE also catalyzes the formation of homocysteine persulfide from homocystine. Nevertheless, these reactions are limited *in vivo* [33,35,37,38,39,158]. Disulfides can also suffer a β-elimination process under alkaline conditions yielding persulfide, a dehydroalanine (2-aminoacrylate) derivative, and water [159,160,161].

## 6. Reactivity of Persulfides with Oxidants

### 6.1. Two-Electron Oxidation

**Scheme 2:**
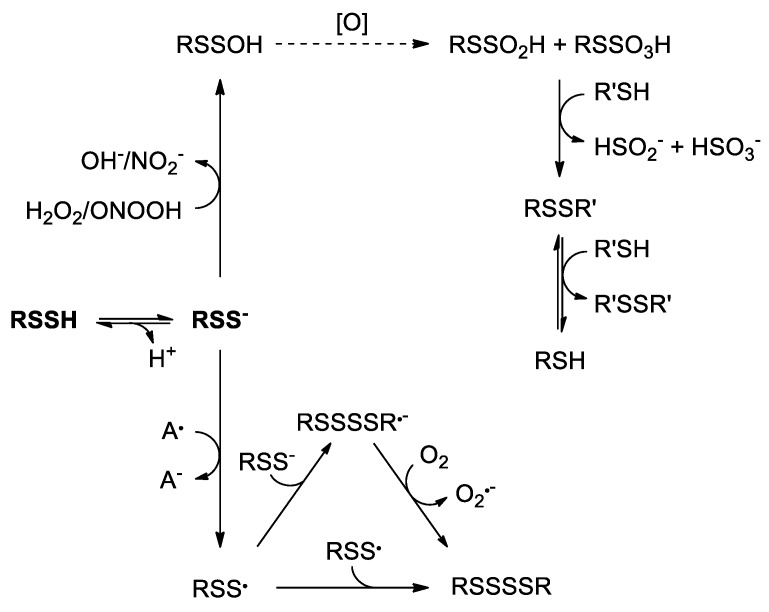
Persulfide oxidation by reactive species. A• represents a one-electron oxidant. The dashed lines represent processes that are accomplished in more than one step. Protons are sometimes omitted for simplicity.

The enhanced nucleophilicity of persulfides versus thiols is also evidenced by the reactions with two-electron oxidizing electrophiles such as H_2_O_2_ and peroxynitrite. Though the reducing abilities of persulfides have not been fully studied, they seem to be better reductants than the corresponding thiols [162]. The reaction of peroxynitrite with albumin persulfide is one order of magnitude faster ((1.2 ± 0.4) × 10^4^ M^–1^ s^–1^ at 20 °C) than the reduced protein at pH 7.4 ((2.7 ± 0.3) × 10^3^ M^–1^ s^–1^) [28]. Furthermore, it was observed that, under certain conditions, H_2_O_2_ did not react with glutathione but rapidly reacted with glutathione persulfide [33]. Analogously to the reaction of H_2_O_2_ with thiols that forms sulfenic acid (RSOH), the reaction with persulfides is expected to produce perthiosulfenic acid (RSSOH). In fact, density functional theory (DFT) calculations predicted that RSSH can be readily oxidized to RSSOH. The reaction of H_2_O_2_ with the persulfide model ethyldisulfane (Et-SSH) to form Et-SSOH was ~6.2 kcal/mol more favorable compared to the oxidation of the corresponding thiol, ethanethiol (Et-SH) to Et-SOH. These calculations also predicted that RSOH and RSSOH are reactive towards dimedone to form stable products. Moreover, the oxidation of cysteine persulfide residues to perthiosulfenic acid was observed in tyrosine kinases such as epidemial growth factor receptor (EGFR) in response to NADPH oxidase activation [163]. In the presence of excess oxidant, the perthiosulfenic acid could be oxidized to perthiosulfinic and perthiosulfonic acids (RSSO_2_H and RSSO_3_H) [20,163]. These species have been detected in papain, albumin and glutathione peroxidase [28,125,151]. It is noteworthy that while sulfinic and sulfonic acids (RSO_2_H and RSO_3_H) are generally considered irreversible modification products, RSSO_2_H and RSSO_3_H can be reduced to thiols by common reductants such as dithiothreitol and enzymes such as thioredoxin, and they can therefore allow the restoration of the reduced protein. Thus, as it was previously mentioned, persulfides could protect protein thiols [29,45,46,153]. The reactions of persulfides with two-electron oxidants are summarized in Scheme 2.

### 6.2. One-Electron Oxidation

Persulfides have enhanced one-electron reducing capability compared to thiols and H_2_S [164]. Firstly, persulfides have a lower S-H bond dissociation energy (70 kcal/mol) than thiols and H_2_S (92 kcal/mol) [165]. Secondly, the one-electron reduction potential of persulfides (*E°’*(RSS•/RSS^–^) = +0.68 V) is lower than those of thiols (*E°’*(RS•, H^+^/RSH) = +0.96 V) and H_2_S (*E°’*(S•^–^, H^+^/HS^–^) = +0.91 V) [87]. Finally, the resonance stabilization energy of RSS• is higher than that of RS•, as the unpaired electron in RSS• is delocalized between the two sulfur atoms [124,164]. Hence, compared to thiols and H_2_S, persulfides can be oxidized by weaker oxidants; the one-electron oxidation products RSS• are less oxidizing than RS• and HS•, react more slowly, and possess an inherent stability. In summary, persulfides constitute better reductants than thiols and H_2_S.

The reaction with one-electron oxidants gives rise to RSS• and can occur through hydrogen atom or electron transfer mechanisms [164]. Persulfides can be oxidized by ferric ions and cannot oxidize ferrous ions [166]. Ferricyanide [47], ferric cytochrome *c* [126,149,167], and metmyoglobin [47] can oxidize persulfides. RSS• is also formed in the reactions with carbon centered radicals [124], peroxyl radicals [164,168], and the nitroxide TEMPOL [47]. These oxidants react more slowly or not at all with the corresponding thiols, exemplifying the better reductant character of persulfides. For instance, TEMPOL and metmyoglobin can be reduced by *N*-methoxycarbonyl penicillamine persulfide but not by an analogous thiol, *N*-acetyl penicillamine [47]. Furthermore, glutathione persulfide was reported to efficiently reduce ferric cytochrome *c*, while glutathione and H_2_S only slowly reduced it [126]. A theoretical study of the reactions of HO• towards a set of persulfides proposed that they could occur through disulfide bond cleavage. In this scenario, RSS• would not be formed [169].

Perthiyl radicals rapidly decay forming tetrasulfide species (RSSSSR) with rate constants of 1–6 × 10^9^ M^–1^ s^–1^ [47,168,170]. In fact, dialkyl tetrasulfide was photolytically cleaved to produce the corresponding RSS•, but dimerization rapidly occurred [168]. The efficiency of this reaction could explain why some thermodynamically unfavorable RSS• formation reactions do occur, such as those of TEMPOL and CH_3_SSH [47], *N*-methoxycarbonyl penicillamine persulfide and ferric cytochrome *c* [87,149], or RSS^–^ and O_2_ [171,172]. Moreover, the resulting RSSSSR can decompose to form RSSR and S_n_ [20].

Perthiyl radicals can react with RSS^–^ to give the intermediate RSSSSR•^–^ that reacts with O_2_ to yield O_2_•^–^ and RSSSSR. However, RSSSSR•^–^ have not been experimentally evidenced [173]. Analogously, RSS• and RS^–^ react producing RSSSR•^–^ [164]. The reactions are summarized in Scheme 2.

Perthiyl radicals, generated via radiolytic reduction of dialkyl trisulfide, were initially reported to react with O_2_ with a rate constant of 5.1 × 10^6^ M^–1^ s^–1^. Such reaction was proposed to form RSSOO• species with subsequent formation of inorganic sulfate [170], which is analogous to thiyl radicals that initially form RSOO• in reversible processes [174,175,176,177]. However, more recent studies suggest that RSS• and O_2_ do not react. Photolytically produced RSS• exhibited the same decay in absence than in presence of O_2_ [168]; besides, oxygen consumption was not altered by addition of perthiyl radicals [47]. Furthermore, computational analysis showed that the reaction between RSS• and O_2_ is endergonic by 16 kcal/mol [47].

The reaction of RS• and NO• to form S-nitrosothiols (RSNO) has been deeply studied [178]. The expected product from the reaction of RSS• with NO• would be S-nitrosopersulfide (RSSNO); however, these radicals do not appear to react. Experimental approaches to synthesize RSSNO failed, with formation of NO• and tetrasulfide. In addition, a solution generating RSS• did not consume NO• [47]. Considering the weakness of the S-N bond determined by computational analyses (3–4 kcal/mol) and the high stability of the radicals, RSSNO is unlikely to exist at room temperature [47,179].

To conclude, persulfides are excellent one-electron reductants. In these reactions, relatively stable perthiyl radicals are formed and can undergo different reactions that ultimately yield RSSSSR.

## 7. Conclusions

In the previous sections, we have summarized the properties of hydrogen sulfide and persulfides, with a focus on redox aspects. A rationalization of the interactions of these species with biologically-relevant reactive oxidants requires an understanding of the kinetics of the processes as well as the elucidation of the fates of the oxidation products formed. While it is becoming clear that direct antioxidant scavenging roles cannot be attributed to H_2_S except under exceptionally high concentrations, many aspects are yet to be understood about persulfides and their rich biochemistry, including their reactions with oxidants.
